# Dietary fiber and prevalence of abdominal aortic calcification in the United States (from the national health and nutrition examination survey data [2013–2014])

**DOI:** 10.1186/s12937-022-00782-0

**Published:** 2022-05-06

**Authors:** YuJiao Sun, HuanRui Zhang, Wen Tian

**Affiliations:** Department of Geriatrics, The First Affiliated Hospital of China Medical University, No.155 Nanjing North Street, Shenyang, 110001 China

**Keywords:** Vascular calcification, Abdominal aortic calcification, Dietary fiber, Nutrition, NHANES

## Abstract

**Background:**

Abdominal aortic calcification (AAC) is recognized as a valuable predictor of cardiovascular diseases (CVDs). Dietary fiber is strongly correlated with CVDs. However, the effect of dietary fiber on AAC in the population is not well understood.

**Objective:**

To assess the relationship between dietary fiber intake and AAC in the US adult population.

**Methods:**

A total of 2671 individuals with both dietary fiber intake and AAC score data were enrolled from the 2013–2014 National Health and Nutrition Examination Survey (NHANES), a cross-sectional health examination in the US. Multinomial logistic regression was used to calculate the odds ratio (OR), with 95% confidence interval (CI). To reveal the relationship between dietary fiber intake and AAC, restricted cubic spline was also applied.

**Results:**

Out of the total participants, 241 (9%) had severe AAC and 550 (20%) had mild-moderate AAC. Multinomial logistic regression indicated that higher intake of dietary fiber was associated with lower risk of severe AAC, but not with lower risk of mild-moderate AAC. For every one standard deviation increase (9.4 g/day) in dietary fiber intake, the odds of severe AAC were reduced by 28% [OR 0.72 (95% CI, 0.57–0.90), *p* = 0.004], after adjusting for confounding factors. Dose–response relationship revealed that dietary fiber intake was negatively correlated with severe AAC (*p* for linear < 0.001, *p* for nonlinear = 0.695).

**Conclusions:**

Dietary fiber intake was negatively associated with severe AAC, and showed a dose–response relationship in US adults.

## Introduction

Aortic calcification is known to be significantly associated with aortic stiffening, atherosclerosis, cardiovascular diseases (CVDs) and mortality [1]. Contrary to classic notion that vascular calcification is irreversible, growing evidence indicates that it is a regulated and reversible process [2]. Therefore, further investigation of the potential factors influencing arterial calcification will provide new therapeutic targets for CVDs.

Dietary fiber, generally regarded as a beneficial dietary nutrient, has been demonstrated to be beneficial for various diseases, such as arterial stiffening, hypertension and CVDs [3–5]. A recent study found that dietary fiber intake could inhibit the progression of vascular calcification in the early stage of chronic kidney disease in rats [6]. However, the relationship between dietary fiber and aortic calcification has not been clarified in the population. In the present study, we collected the data from the National Health and Nutrition Examination Survey (NHANES), assessed the relationship between dietary fiber and abdominal aortic calcification (AAC), and analyzed their dose–response relationship.

## Methods

### Data source and participants

Data were extracted from the 2013–2014 NHANES database, a cross-sectional health examination conducted by the National Center for Health Statistics (NCHS) in the US. The NHANES protocol were approved by the NCHS Ethics Review Board, and informed consent forms were signed by all participants. Among a total of 10,175 participants in the 2013–2014 cohort, AAC was evaluated in participants ≥ 40 years. Finally, we enrolled 3140 participants with valid information on AAC scores. After further excluding those without complete information about dietary fiber or potential covariates, we included 2671 individuals in the subsequent analysis.

### Dietary fiber intake

The independent variable was dietary fiber intake (g), which was obtained from the 24-h recall survey. The Automated Multiple-Pass Method was used to collect dietary data, including the types and amounts of all foods and beverages consumed during the 24-h period prior to the interview, which were used to estimate intakes of energy and nutrients [7]. Dietary fiber intake was calculated according to the US Department of Agriculture (USDA) Food and Nutrient Databases for Dietary Studies (FNDDS) (https://www.cdc.gov/nchs/tutorials/dietary/SurveyOrientation/ResourceDietaryAnalysis/intro.htm). The first 24-h recall survey was conducted in the Mobile Examination Center (MEC) and the second was collected by telephone 3–10 days later. Long-run average nutrient intakes could be estimated using two days of intake data for each participant in the US. The dietary fiber intake was calculated as an average of two days dietary recall data if two days data was available. Otherwise, single dietary recall was used.

### AAC evaluation

The primary outcome variable was AAC, which was obtained from the lumbar vertebrae L1-L4 using dual-energy X-ray absorptiometry (DXA) and calculated using Kauppila score system for AAC [8]. DXA showed high specificity and sensitivity for detecting AAC in the lateral lumbar spine images, was inexpensive and had reduced radiation exposure (A-C) [8–10]. DXA scans were conducted on eligible survey participants aged ≥ 40 years, no pregnancy, no history of barium use in past seven days and < 450 pounds of body weight. The Kauppila score ranged from 0 to 24. A Kauppila score > 6 is predictor for significant aortic calcification, and has been used as a cutoff value in previous studies [11, 12]. In this study, individuals were divided into three groups: no AAC (score = 0), mild-moderate AAC (0 < score ≤ 6), and severe AAC (score > 6).

### Potential risk factors for AAC

We collected demographic information, co-morbidities, smoking status, total cholesterol (TC), high density lipoprotein cholesterol (HDL-C), albumin, creatinine, total calcium, phosphorus, white blood cells (WBC), total 25-hydroxyvitamin D, and caloric intake data of the participants. Body mass index (BMI) was calculated as body weight (Kg) divided by the square of height (m). Hypertension was defined as the average of three measurements of resting blood pressure ≥ 140/90 mmHg, self-reported diagnosis of hypertension, or taking antihypertensive medications. Diabetes mellitus (DM) was defined as a fasting glucose ≥ 126 mg/dL, self-reported diagnosis of diabetes, or taking hypoglycemic agent or insulin. Smoker was defined as someone who smoked at least 100 cigarettes in a lifetime. The lipid profile level was presented as a ratio of TC to HDL-C.

### Statistical analysis

R version 4.0.3 was used for analyses, with incorporation of weights, primary sampling unit and strata supplied by NHANES. Continuous variables were summarized as means (standard deviation, SD) and compared using ANOVA. Categorical variables were presented by counts and percentages, and compared using Pearson’s c^2^ test. The odds ratio (OR) with 95% confidence interval (CI) for mild-moderate and severe AAC were evaluated by multinomial logistic regression, using the lowest quartiles of dietary fiber intake as the reference. Crude was not adjusted for any covariates. Model 1 was adjusted for age, gender and ethnicity. Model 2 was adjusted for Model 1 covariates plus BMI, education, DM, hypertension, smoking, TC/HDL-C, albumin, creatinine, total calcium, phosphorus, WBC, total 25-hydroxyvitamin D, and caloric intake. Dose–response relationship between dietary fiber and severe AAC was depicted using restricted cubic spline. A two-sided *p*-value < 0.05 was considered significant.

## Results

### Baseline characteristics of the study participants

**Fig. 1:**
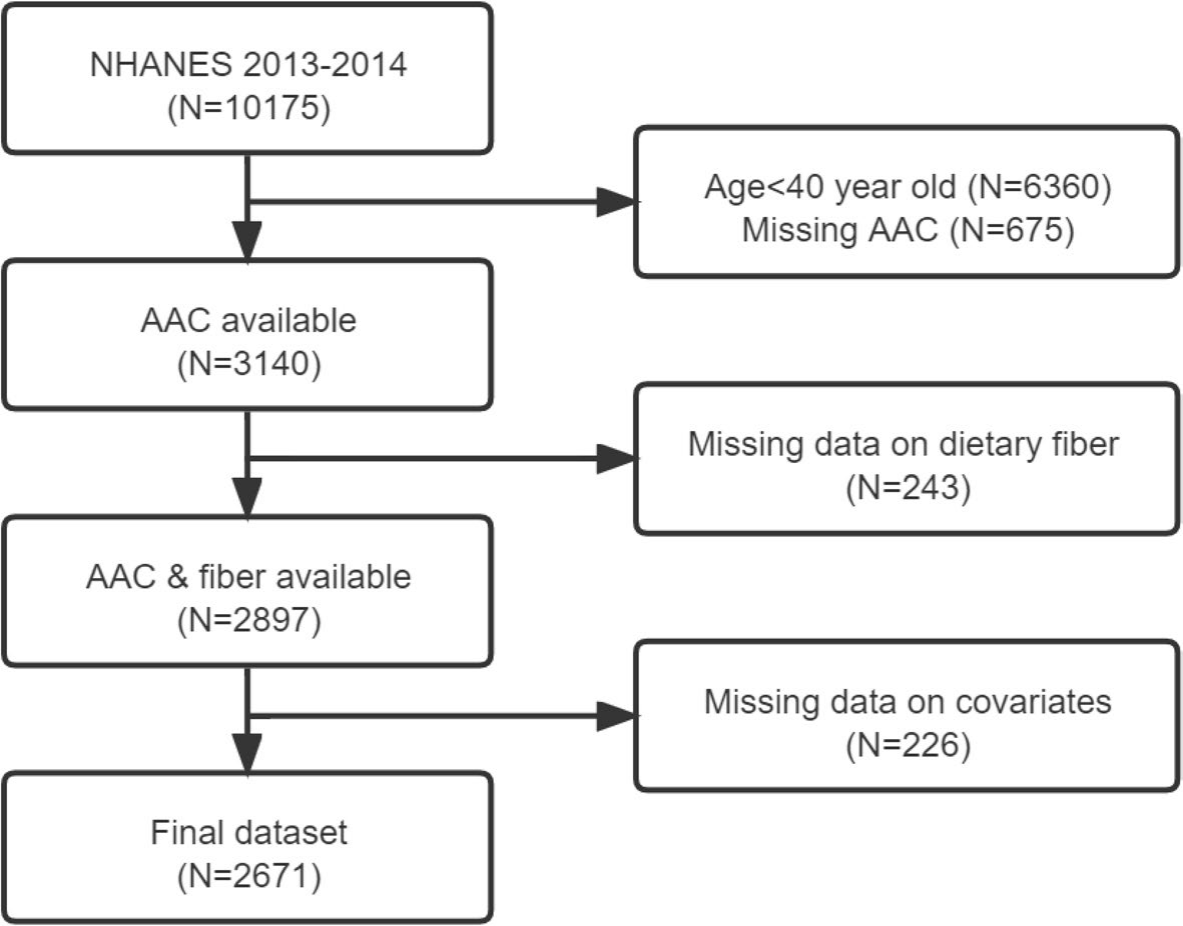
Flow chart of participant selection process

After the application of inclusion and exclusion criteria, 2671 eligible individuals were recruited into the study (Fig. 1). All participants were divided into three groups: no AAC (score = 0), mild-moderate AAC (0 < score ≤ 6), and severe AAC (score > 6). The baseline characteristics are shown in Table 1. Out of the total participants, 241 (9%) had severe AAC and 550 (20%) had mild-moderate AAC. Compared to participants without AAC, those with AAC were older, had a higher prevalence of DM and hypertension, and were smokers. They also had higher values of serum and total 25-hydroxyvitamin D, but lower intake of dietary calories and fiber.

**Table 1:** Participant characteristics by AAC groups

Variables	Total	No AAC (score = 0)	Mild–moderate AAC (0 < score ≤ 6)	Severe AAC (score > 6)	*P* value
	(*n* = 2671)	(*n* = 1880)	(*n* = 550)	(*n* = 241)	
Age, n (%)					< 0.001
40–49	742 (29.7)	630 (35.2)	104 (20.6)	8 (2.8)	
50–59	678 (29.2)	536 (32.0)	122 (26.7)	20 (10.0)	
60–69	674 (23.0)	467 (22.5)	155 (25.4)	52 (22.1)	
≥ 70	577 (18.0)	247 (10.3)	169 (27.3)	161 (65.0)	
Sex, n (%)					0.588
Men	1291 (48.2)	898 (48.1)	278 (50.1)	115 (44.5)	
Women	1380 (51.8)	982 (51.9)	272 (49.9)	126 (55.5)	
Ethnicity, n (%)					0.022
Non-Hispanic White	1220 (72.4)	782 (70.6)	279 (76.0)	159 (80.4)	
Non-Hispanic Black	509 (9.6)	388 (10.4)	92 (8.1)	29 (6.0)	
Mexican American/Hispanic	605 (11.3)	467 (12.5)	107 (8.9)	31 (6.6)	
Other	337 (6.6)	243 (6.5)	72 (7.1)	22 (7.0)	
Body mass index, kg/m2	28.57 (5.51)	28.84 (5.78)	27.98 (4.84)	27.58 (4.22)	0.001
Education, n (%)					0.059
Less than high school	588 (14.7)	409 (14.0)	119 (14.7)	60 (21.7)	
Higher than high school	2083 (85.3)	1471 (86.0)	431 (85.3)	181 (78.3)	
Diabetes mellitus, n (%)					< 0.001
Absence	2071 (82.1)	1515 (84.9)	413 (80.2)	143 (60.9)	
Presence	600 (17.9)	365 (15.1)	137 (19.8)	98 (39.1)	
Hypertension, n (%)					< 0.001
Absence	1229 (49.6)	976 (55.5)	209 (39.3)	44 (21.3)	
Presence	1442 (50.4)	904 (44.5)	341 (60.7)	197 (78.7)	
Smoking, n (%)					< 0.001
Absence	1439 (54.6)	1083 (58.9)	262 (45.8)	94 (37.7)	
Presence	1232 (45.4)	797 (41.1)	288 (54.2)	147 (62.3)	
Total cholesterol /High density lipoprotein cholesterol	3.86 (1.37)	3.84 (1.38)	3.98 (1.37)	3.76 (1.20)	0.185
Serum albumin, g/dL	4.25 (0.30)	4.26 (0.30)	4.25 (0.31)	4.20 (0.29)	0.065
Serum creatinine, mg/dL	0.92 (0.39)	0.90 (0.30)	0.95 (0.59)	1.03 (0.50)	0.002
Serum total calcium, mg/dL	9.46 (0.36)	9.44 (0.36)	9.49 (0.36)	9.48 (0.36)	0.361
Serum phosphorus, mg/dL	3.80 (0.56)	3.79 (0.56)	3.77 (0.56)	3.90 (0.58)	0.094
White blood cells, 1000 cells/uL	7.19 (2.23)	7.13 (2.32)	7.32 (1.97)	7.38 (2.03)	0.17
Total 25-hydroxyvitamin D, nmol/L	75.30 (29.50)	74.54 (29.28)	75.34 (29.51)	82.20 (30.75)	0.018
Caloric intake, kcal/day	2014.50 (792.89)	2036.63 (806.06)	2021.58 (782.95)	1790.50 (651.04)	0.014
Dietary fiber, g	17.24 (8.85)	17.65 (9.16)	16.64 (8.08)	15.00 (7.37)	0.004

All values represented are weighted means ± standard error, or counts (weighted prevalence)

### Relationship between dietary fiber and AAC

Multinomial logistic regression indicated that higher intake of dietary fiber (continuous variable) was associated with lower risk of severe AAC, but not with lower risk of mild-moderate AAC (Table 2). For every one SD increase (9.4 g/day) in the intake of dietary fiber, the OR of severe AAC decreased by 28% in model 2 (OR [95% CI], 0.72(0.57–0.90), *p* = 0.004).

**Table 2:** Multinomial logistic regression models of dietary Fiber intake and AAC groups

Fiber quartile							
	Fiber increase/SD		Q1 (≤ 10.95)	Q2 (10.96–15.60)	Q3 (15.61–21.77)	Q4 (> 21.77)	*P* for trend
Mild-moderate AAC vs No AAC							
Crude	0.93 (0.85–1.03)		Ref	1.10 (0.84–1.44)	1.14 (0.88–1.49)	0.84 (0.64–1.11)	0.286
Model1	0.93 (0.83–1.03)		Ref	1.03 (0.78–1.35)	1.05 (0.79–1.38)	0.80 (0.60–1.07)	0.171
Model2	0.90 (0.80–1.02)		Ref	1.08 (0.90–1.29)	1.09 (0.92–1.28)	0.79 (0.67–0.93)**	0.207
Severe AAC vs No AAC							
Crude		0.69 (0.58–0.81)***	Ref	1.03 (0.73–1.45)	0.65 (0.44–0.95)*	0.51 (0.34–0.76)**	< 0.001
Model1		0.65 (0.54–0.79)***	Ref	0.85 (0.58–1.25)	0.51 (0.34–0.79)**	0.47 (0.30–0.73)**	< 0.001
Model2		0.72 (0.57–0.90)**	Ref	1.16 (0.88–1.53)	0.70 (0.54–0.92)**	0.71 (0.56–0.90)**	0.060

Crude adjusted for no variable. Model 1 adjusted for age, sex and ethnicity (non-Hispanic White, non-Hispanic black, Mexican American/Hispanic, and other races). Model 2 further adjusted for body mass index (continuous), education (less than high school and higher than high school), diabetes mellitus (yes and no), hypertension (yes and no), smoking (yes and no), total cholesterol /high density lipoprotein cholesterol (continuous), serum albumin (continuous), serum creatinine (continuous), serum total calcium (continuous), serum phosphorus (continuous), white blood cells (continuous), total 25-hydroxyvitamin D (continuous), and Caloric intake (continuous). * *P* < 0.05; ** *P* < 0.01; *** *P* < 0.001

Table 2 also shows the association of the quartiles of dietary fiber level with the AAC. Compared with the lowest quartile of dietary fiber intake in Model 2, the participants in the highest quartile were associated with lower odds for mild-moderate AAC [Quartile 4 vs. Quartile 1: OR 0.79 (95% CI, 0.67–0.93), *p* = 0.004], and higher intake of dietary fiber was associated with lower odds for severe AAC [Quartile 4 vs. Quartile 1: OR, 0.71 (95% CI, 0.56–0.90), *p* = 0.005; Quartile 3 vs. Quartile 1: OR 0.70 (95% CI, 0.54–0.92), *p* = 0.010].

As shown in Table 3, age, ethnicity, BMI, DM, hypertension, smoking, creatinine, phosphorus, and WBC remained significant in model 2. Participants aged ≥ 70 years had 29 times higher risk of severe AAC compared to those aged < 49 years (*p* < 0.001). Compared to Non-Hispanic White population, the other ethnicities had lower risk of severe AAC (all *p* < 0.001). Presence of DM, hypertension, and smoking was associated with higher risk of severe AAC (all *p* < 0.001). The risk of severe AAC was positively correlated with the values of creatinine, phosphorus, and WBC, but negatively correlated with BMI (all *p* < 0.05).

**Table 3:** Multinomial logistic regression model of dietary Fiber intake and AAC groups in Model2

Variable	Mild–moderate AAC vs No AAC		Severe AAC vs No AAC	
	OR (95% CI)	*P* value	OR (95% CI)	*P* value
Fiber increase/SD	0.90 (0.80–1.02)	0.104	0.72 (0.57–0.90)	0.004
Age, (vs 40–49)				
50–59	1.31 (1.10–1.55)	0.002	2.44 (2.29–2.60)	< 0.001
60–69	1.76 (1.51–2.06)	< 0.001	6.11 (5.11–7.30)	< 0.001
≥ 70	3.59 (3.07–4.20)	< 0.001	34.44 (28.37–41.8)	< 0.001
Sex, (vs man)	1.12 (0.91–1.38)	0.276	1.15 (0.99–1.35)	0.077
Ethnicity, (vs Non-Hispanic White)				
Non-Hispanic Black	0.68 (0.55–0.83)	< 0.001	0.36 (0.34–0.38)	< 0.001
Mexican American/Hispanic	0.80 (0.66–0.97)	0.026	0.49 (0.44–0.55)	< 0.001
Other	0.97 (0.79–1.19)	0.798	0.68 (0.65–0.72)	< 0.001
Body mass index, kg/m^2^	0.95 (0.93–0.97)	< 0.001	0.92 (0.89–0.95)	< 0.001
Education, (vs less than high school)	1.06 (0.84–1.34)	0.615	0.98 (0.80–1.20)	0.840
Diabetes mellitus, (vs no)	1.29 (1.03–1.62)	0.027	2.45 (1.97–3.05)	< 0.001
Hypertension, (vs no)	1.47 (1.20–1.80)	< 0.001	2.61 (2.28–2.99)	< 0.001
Smoking, (vs no)	1.42 (1.16–1.74)	0.001	2.31 (1.74–3.05)	< 0.001
Total cholesterol /High density lipoprotein cholesterol	1.09 (1.01–1.18)	0.021	1.05 (0.93–1.18)	0.467
Serum albumin, g/dL	0.90 (0.83–0.97)	0.008	1.56 (1.44–1.68)	< 0.001
Serum creatinine, mg/dL	1.17 (0.96–1.43)	0.124	1.28 (1.06–1.56)	0.012
Serum total calcium, mg/dL	1.21 (1.11–1.31)	< 0.001	0.83 (0.73–0.94)	0.003
Serum phosphorus, mg/dL	0.90 (0.75–1.07)	0.239	1.34 (1.07–1.68)	0.012
White blood cells, 1000 cells/uL	1.00 (0.96–1.05)	0.936	1.08 (1.01–1.16)	0.033
Total 25-hydroxyvitamin D, nmol/L	1.00 (1.00–1.00)	0.806	1.00 (0.99–1.00)	0.507
Caloric intake, kcal/day	1.00 (1.00–1.00)	0.050	1.00 (1.00–1.00)	0.768

**Fig. 2:**
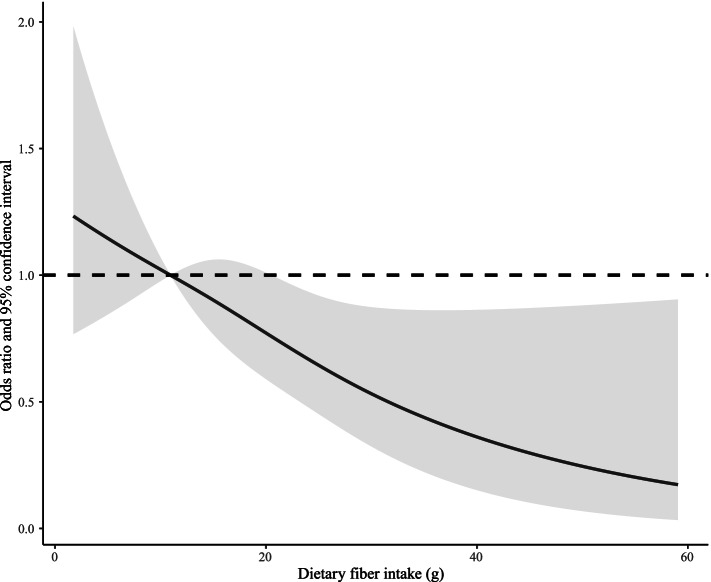
Dose–response relationship between dietary fiber intake and severe AAC (p for linear < 0.001, p for nonlinear = 0.695), using the cutoff value of lowest quartile (Q1) of dietary fiber intake (10.95 g) as the reference. AAC24 score > 6 was defined as severe AAC. The restricted cubic spline model was adjusted by age, gender, ethnicity, BMI, education, DM, hypertension, smoking, TC/HDL-C, albumin, creatinine, total calcium, phosphorus, WBC, total 25-hydroxyvitamin D, and caloric intake

In the restricted cubic spline model, participants with AAC score > 6 were defined as severe AAC, using AAC score ≤ 6 as the reference. Dose–response relationship revealed that intake of dietary fiber was negatively correlated with severe AAC (p for linear < 0.001, p for nonlinear = 0.695), using the lowest quartile (Q1) of dietary fiber intake (10.95 g) as the reference (Fig. 2).

## Discussion

Based on 2013–2014 NHANES, the present study showed that dietary fiber intake was negatively associated with severe AAC in a dose–response manner, which suggested that dietary fiber played a protective role in severe AAC. After adjusting for potential confounding factors, dietary fiber was an independent protective factor for severe AAC. The odds of suffering severe AAC decreased by 28% for every one standard deviation (9.4 g/day) increase in dietary fiber.

Dietary factors are strongly correlated with risk factors for CVDs and mortality [13]. Healthy dietary strategies have been recommended for decreasing the risk of CVDs [14]. Several studies have found that diet has a significant influence on AAC. A study on community-dwelling adults suggested that dietary quality assessed by AHEI-2010 was an independent protective factor of AAC, and the risk of AAC was lower with higher dietary quality [15]. A study of 3718 participants found that people with Mediterranean dietary pattern had attenuated progression and severity of coronary artery calcification [16]. Another study analyzed the relationships between some food groups and AAC, and found that apple intake was an independent protective factor for AAC in older women, each additional 50 g/day apple intake resulted in a 24% lower OR of severe AAC [17]. A study found that cruciferous vegetable intake was negatively correlated with AAC [18]. Other studies have reported the effects of dietary nutritional intake, such as magnesium and zinc, on coronary artery calcification and AAC [19, 20]. Dietary fiber is a very important nutriment, which plays a vital role in maintaining our health. However, the effect of dietary fiber on AAC in the population is not well understood and deserves further investigation.

A meta-analysis of ten long-term follow-up studies indicated that dietary fiber intake was negatively correlated with the risk of CVDs [RR:0.84 (95% CI, 0.70–0.99)], but further analysis showed that an extra 10 g/day of dietary fiber intake failed to significantly reduce the risk of CVDs [RR:1.0 (95% CI 0.88–1.13)] [21]. Another systematic review, focused on the association of dietary fiber with CVDs and potential dose–response relationship [22], demonstrated that for each extra 7 g/day intake of dietary fiber, the risk of CVDs decreased by 9% [RR:0.91 (95% CI 0.87 to 0.94)] [22]. A recent meta-analysis of 15 prospective cohort studies revealed that an increased intake of dietary fiber had reduced cardiovascular death by 23%, and an increase of 10 g/day dietary fiber intake could decrease the mortality of CVDs by 9% [RR:0.91 (95% CI: 0.88–0.94)] [23]. Some clinical studies validated the role of dietary fiber on the risk factors of CVDs and demonstrated that dietary fiber significantly improved hypertension, lipid profile, diabetes and metabolic syndrome [5, 24–26]. Previous investigations have proven that dietary fiber was associated with the risk of CVDs. However, the relationship between dietary fiber and AAC, a subclinical marker of atherosclerosis and CVD risk, remains largely unknown.

AAC is widely recognized as a better predictor of atherosclerosis and CVDs than Framingham risk score [27]. The MESA study found that AAC, but not coronary artery calcification, was closely related to CVDs and all-cause mortality [28]. The incidence of AAC in the Framingham heart study was 22.4% in males and 16.4% in females under 45 years of age, and was as high as 100% in both males and females over 75 years of age [29]. However, there are no published prospective trials specifically designed for testing the effect of dietary fiber on AAC in humans. The present study aimed to evaluate the association of dietary fiber and AAC in the population, and showed that dietary fiber is an independent protective factor for severe AAC, and has a linear negative dose–response relationship with severe AAC. The results of the present study are consistent with previous studies on the relationship between dietary fiber and CVDs [21–23]. The findings on the role of dietary fiber on AAC might provide a new perspective on the prevention of CVDs.

We hypothesized that several underlying mechanisms might explain the association of dietary fiber with AAC. First, imbalanced phosphorous metabolism plays a major role in vascular calcification [30]. Dietary fiber was reported to inhibit the progression of vascular calcification by improving repeated phosphorus fluctuations in early stage chronic kidney disease in rats [7]. Second, some studies indicated that inflammation participated in the pathophysiological process of vascular calcification. Inflammatory cytokines stimulated vascular smooth muscle cell calcification, which could be inhibited by reducing secretion of inflammatory cytokines [31, 32]. Higher dietary fiber intake was associated with lower level of C-reactive protein, a classical inflammatory marker [33, 34]. In addition, dietary fiber increased the level of anti-inflammatory factors, which might contribute to relieve inflammation [35]. Moreover, the deposition of lipids on arterial vessel walls promoted vascular intimal calcification [30]. Studies showed that dietary fiber intake was beneficial to lower cholesterol [26, 30, 35]. Finally, intestinal microbiota influenced the occurrence and development of CVDs [36]. Dietary fiber plays an important role in the composition and function of human intestinal microbiota [37]. The effect of dietary fiber on CVDs may be partly achieved through regulating intestinal microbiota. The above aspects might be potential mechanisms to explain the association of dietary fiber with AAC, but the actual mechanisms remain unclear and deserve further investigation.

This study had some limitations. The present cross-sectional study could not establish a causal link between dietary fiber and AAC. Dietary fiber data were obtained from dietary recall interviews, which have risk of self-report bias. The participants were from NHANES of nationally representative, non-institutionalized US general population, but the results of this study may not be applicable to people of other races and with specific diseases.

## Conclusion

The dietary fiber intake was negatively associated with severe AAC, and high dietary fiber was an independent protective factor of severe AAC. Dietary fiber had important value in severe AAC, which suggested that dietary fiber might contribute to reduce the risk of vascular calcification and CVDs. So dietary fiber might be a new strategy for preventing vascular calcification, thereby reducing the risk of cardiovascular diseases. Further studies are needed to determine the causal relationship between dietary fiber and severe AAC as well as the underlying mechanisms.

## Data Availability

Data are available on the NHANES website.
